# Tortuosity in non-atherosclerotic vascular diseases is associated with age, arterial aneurysms, and hypertension

**DOI:** 10.1186/s13023-024-03231-9

**Published:** 2024-06-07

**Authors:** Xhyljeta Luta, Fabio Zanchi, Marco Fresa, Enrica Porceddu, Sanjiv Keller, Judith Bouchardy, Sébastien Déglise, Salah Dine Qanadli, Matthias Kirsch, Grégoire Wuerzner, Andrea Superti-Furga, Giacomo Buso, Lucia Mazzolai

**Affiliations:** 1 Department of Angiology, University Hospital of Lausanne (CHUV), Lausanne, Switzerland; 2 Department of Radiology, University Hospital of Lausanne (CHUV), Lausanne, Switzerland; 3 Department of Cardiology, University Hospital of Lausanne (CHUV), Lausanne, Switzerland; 4 Department of Vascular Surgery, University Hospital of Lausanne (CHUV), Lausanne, Switzerland; 5 Department of Cardiac Surgery, University Hospital of Lausanne (CHUV), Lausanne, Switzerland; 6 Department of Nephrology and Hypertension, University Hospital of Lausanne (CHUV), Lausanne, Switzerland; 7 Department of Genetic Medicine, University Hospital of Lausanne (CHUV), Lausanne, Switzerland; 8 Riviera-Chablais Hospital, University of Lausanne, Lausanne, Switzerland

**Keywords:** Rare vascular diseases, Non-atherosclerotic vascular diseases, Arterial tortuosity, Tortuosity index, Computed tomography angiography

## Abstract

**Background:**

Increased arterial tortuosity has been associated with various cardiovascular complications. However, the extent and role of arterial tortuosity in non-atherosclerotic vascular diseases remain to be fully elucidated. This study aimed to assess arterial tortuosity index (ATI) in patients with non-atherosclerotic vascular diseases and the associated factors.

**Methods:**

This is a retrospective analysis of patients with non-atherosclerotic vascular diseases referred to the Malformation and Rare Vascular Disease Center at the University Hospital in Lausanne (Switzerland). Computed tomography angiography (CTA) images performed between October 2010 and April 2022 were retrieved and the aortic tortuosity index (ATI) was calculated. Patients were classified based on diagnosis into the following groups: arterial dissection & aneurysm, arteritis & autoimmune disease, hereditary connective tissue diseases, and fibromuscular dysplasia (FMD). Univariate and multivariate logistic regression analysis was used to determine potentially relevant predictors of aortic tortuosity.

**Results:**

The mean age upon computed tomography angiography (CTA) was 46.8 (standard deviation [SD] 14.6) years and 59.1% of the patients were female. Mean ATI was higher in patients over 60 years old (1.27), in those with arterial aneurysms (mean: 1.11), and in those diagnosed with hypertension (mean: 1.13). When only patients over 60 years old were considered, those diagnosed with connective tissue diseases had the highest ATI. At multivariate regression analysis, increasing age (*p* < 0.05), presence of arterial aneurysms (*p* < 0.05), and hypertension (*p* < 0.05) were independently associated with ATI.

**Conclusions:**

The ATI may be a promising tool in diagnostic evaluation, cardiovascular risk stratification, medical or surgical management, and prognostic assessment in several non-atherosclerotic vascular conditions. Further studies with longitudinal design and larger cohorts are needed to validate the role of ATI in the full spectrum of vascular diseases.

**Supplementary Information:**

The online version contains supplementary material available at 10.1186/s13023-024-03231-9.

## Background

Rare vascular diseases include a myriad of different pathologies involving blood vessels. Although each disease has low prevalence affecting at most one person in 2000, such conditions can cause distressing symptoms that significantly affect the quality of life of patients and have a major impact on public health overall [1]. Due to their heterogeneity and rare nature, certain physiopathological mechanisms remain poorly understood.

Arterial tortuosity is characterized by the presence of abnormal twists and turns of one or several arteries [2]. The underlying causes of tortuosity are not yet well understood. It could be a compensatory mechanism in patients with cardiovascular risk factors such as hypertension where mechanical factors including blood flow and axial tension could cause structural changes in vessel walls [3, 4]. Previous research has linked arterial tortuosity to older age, hypertension, and atherosclerosis [3, 5]. However, recently, arterial tortuosity has been described as a new feature of several rare vascular diseases [2, 6, 7] that could be associated with aneurysms or dissection of medium-sized arteries including coronary, pulmonary, and peripheral arteries or genetic conditions with SLC2A1 mutation [8–10]. In these cases, other clinical typical craniofacial manifestations (*e.g.,* long face, hypertelorism, and sagging cheeks) and other clinical features including *cutis laxa*, diaphragmatic hernia, and Marfanoid skeletal features can be present [11–13]. With the advancement of imaging technology, various forms of tortuosity have been described including curving/curling, angulation, twisting, looping and kinking vessels [14]. Intriguingly, it has been suggested that the degree of tortuosity could be used to assess the presence and severity of various vascular diseases as well as to determine cardiovascular risk and appropriate surgical timing [2, 7, 15–21].

Previous studies have shown that arterial tortuosity may cause several complications including pulmonary arteries stenosis, focal or multiple stenosis of the aorta, aortic and arterial aneurysms and dissections, ischemic events, and large-vein dilation [22, 23]. However, the presence and role of arterial tortuosity in patients with rare vascular diseases are still unclear.

Considering these gaps in knowledge, the aims of this study were to: (1) estimate the arterial tortuosity index (ATI); (2) evaluate the association between ATI and a series of clinical features; and (3) assess independent predictors of arterial tortuosity in a cohort of patients with non-atherosclerotic vascular diseases.

## Methods

### Study design and setting

This is a retrospective analysis of patients with non-atherosclerotic vascular diseases followed at the Malformation and Rare Vascular Disease Center of Lausanne University Hospital (CHUV, Switzerland) and included in the RAVAD (Registry of rAre VAscular Diseases) registry. The center provides highly specialized medical services in the field of vascular medicine including care for patients with vein, artery, lymphatic, and microcirculation disorders. The RAVAD registry was implemented in July 2017 for both clinical and research purposes (Protocol number: 2016–02005). The main objective of the registry is to identify patients with rare vascular diseases to optimize their care. Additionally, clinical, biological, and imaging data are collected allowing investigation of disease presentation patterns (phenotype) and evolution (characteristics, management, outcome with or without treatment).

### Patient selection and data extraction

**Fig. 1:**
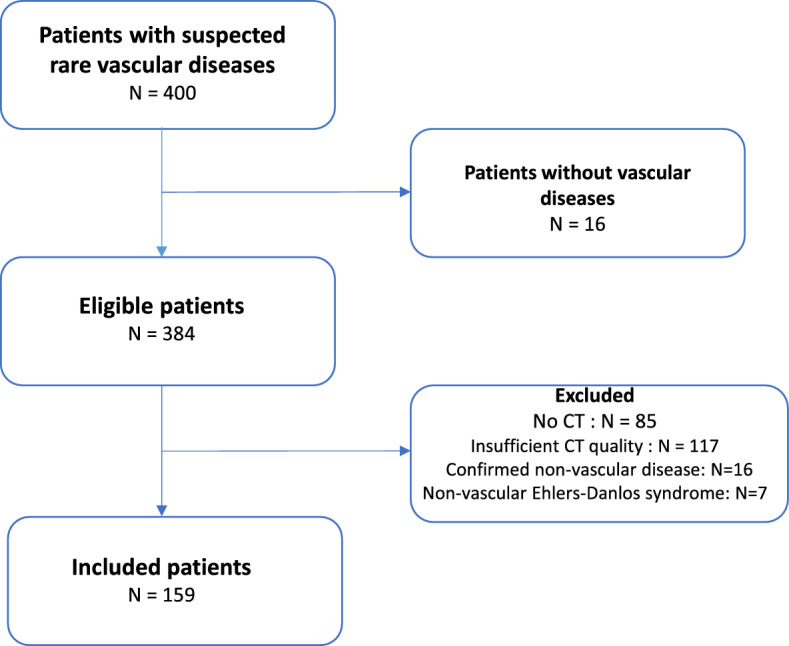
Participant selection process

Figure 1 shows the selection of study participants. We retrospectively analyzed all patients referred to our center between April 2010 and May 2022. Of 400 eligible patients referred for suspected non-atherosclerotic vascular diseases, 32 patients (16 at the start of the study and 16 later once diagnosis was confirmed) had no vascular disease and were excluded. Patients diagnosed with non-vascular Ehlers-Danlos syndrome (*n* = 7) were also excluded, as well as those who did not undergo a contrast-enhanced computed tomography angiography (CTA) (*n* = 85) and those with insufficient CTA quality (*n* = 117).

For each participant, the following socio-demographic and clinical parameters were recorded: sex, age, height (cm), Body Mass Index (BMI), hypertension status, ongoing pharmacological treatment, family history for rare vascular disease, presence of genetic mutations associated with connective tissue disorders, vascular diagnosis, presence and type of arterial lesion, presence of aortic root dilation, presence of joint hyperlaxity, previous vascular intervention.

Vascular diagnosis was classified into four categories: non traumatic arterial dissection & aneurysm, arteritis, autoimmune disease, hereditary connective tissue diseases, and fibromuscular dysplasia (FMD) The first group includes patients with CTA finding of dissection and/or aneurysm of the aorta and/or peripheral arteries not associated with hereditary connective tissue disease. The second group includes patients with various diagnoses of small, medium, and large vessel vasculitis in addition to other systemic autoimmune diseases. The third group includes patients with confirmed diagnoses of Marfan syndrome, Loeys-Dietz syndrome, vascular Ehlers-Danlos syndrome, familial thoracic aortic aneurysms and dissections, aneurysms-osteoarthritis syndrome, and other rare inherited connective tissue diseases with vascular involvement.

For each participant, the presence of arterial lesions was extracted from patient’s medical record. The arterial lesions were then classified as follows: dissection, aneurism, occlusion, and stenosis. Aortic root dilation was defined as a calculated Z-score ≥ 2 standardized to age and body size, as previously described [24]. Joint hyperlaxity was defined as a Beighton score ≥ 5 [25].

Socio-demographic and clinical data were extracted from the electronic patient records and validated by two of the co-authors (M.F., G.B.).

### Imaging procedure

CTA images were obtained using a variety of CT scanners in different centers. Typical acquisition protocol included high resolution images (slice thinness 1.25 mm, adjusted field of view) after a single intravenous iodine contrast agent injection (volume ranging from 80 to 120 ml, rate: 4 ml/second) followed by 40 ml saline injection at the same rate. The volume of data acquired started usually above the aortic arch and ended at the femoral bifurcation.

### Measurement of aortic tortuosity index

Available CTA images were retrieved from the PACS and transferred to a workstation.

**Fig. 2:**
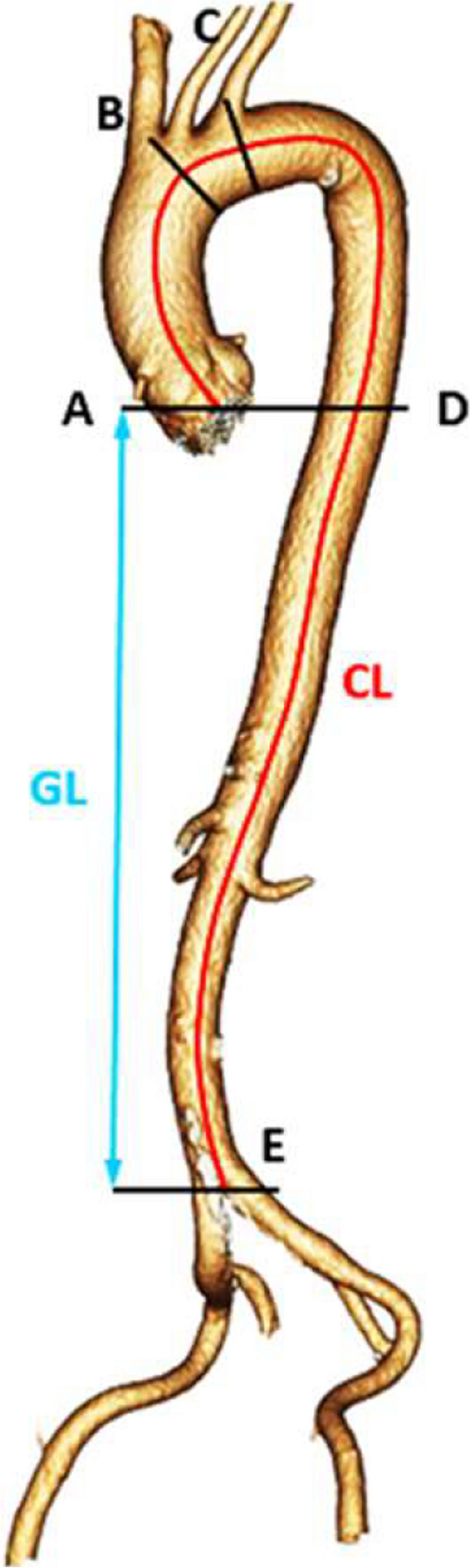
Computed tomography volume rendering of the aorta of a 61-year-old male participant. ATI was calculated as the ratio between the centerline length (CL) and the geometric length (GL) as described by Dougherty & Varro [26]

Details on ATI measurement are described in Fig. 2. The centerline length was defined as the length of the centerline from the annulus to the aortic bifurcation. The geometric length was defined as the vertical distance from the annulus to the aortic bifurcation on a CT volume rendering of the aorta. Volume rendering reconstructions were created using the institution’s PACS (Vue PACS, Carestream Health Inc., Rochester, Illinois, US). The volume rendering images shown in this study were produced using GE Healthcare AW server 3.2 (Chicago, Illinois, US). All patients CTA images were analyzed by the radiologist (F.Z).

### Statistical analysis

We conducted descriptive statistics to summarize the patient sociodemographic and clinical characteristics. We described continuous variables with mean and standard deviation (SD), and categorical variables as counts and percentages. Normal distribution of continuous data was performed using Shapiro–Wilk test. Comparison between the two groups was done with the independent sample t-test for normally distributed data and the Mann–Whitney U test for non-normally distributed data. Categorical variables were compared by Pearson chi-square analysis and Fisher test and ANOVA for multiple comparisons. Simple and multivariable linear regression analyses were performed to investigate potential predictors of arterial tortuosity including age, sex, BMI, lesion type, and hypertension status. Statistical significance was set at < 0.05 for all analysis. Data management and analysis were performed using Stata version 16 (StataCorp, College Station, TX, USA).

## Results

### Demographic and clinical characteristics

In total, 159 patients met the selection criteria and were included in the study. Of these, 59.1% were females and the overall mean age at time of CTA was 46.8 (SD: 14.6) years. The main clinical and demographic characteristics of the patients split by gender and diagnosis are reported in Table 1.

**Table 1:** Comparison of demographic and clinical characteristics between males and females

Characteristics (*n* = 159)	Male (*n* = 65, 40.9%)	Female (*n* = 94, 59.1%)	*p*-value
Age at CT scan (mean, ± SD)	48.1 (15.2)	45.9 (14.2)	0.35
Height (mean, range)	178.3 (8.2)	164.4 (8.0)	0.001
Weight	7 77.6 (12.7)	64.9 (10.6)	0.001
Body Mass Index, kg/m2 (mean, range)	24.6 (16.2–30.9)	24.1 (16.6–31.6)	0.44
**Family history (n, %)**			0.19
Yes	4 (6.2)	12 (12.8)	
No	61 (93.8)	82 (87.2)	
**Confirmed genetic mutation (n, %)**			0.11
Yes	9 (13.8)	23 (24.5)	
No	56 (86.2)	71 (75.5)	
**Z-score (n, %)**			
Z-score ≥ 2	22 (33.8)	16 (17.0)	0.02
Z-score < 2	43 (66.2)	78 (83.0)	
**Beighton score (n, %)**			0.08
Score ≥ 5	1 (3.1)	8 (16.0)	
Score < 5	31 (96.9)	42 (84.0)	
**Diagnosis** (n, %)			
Arterial dissection & aneurysm	27 (41.5)	27 (28.7)	0.27
Arteritis & autoimmune disease	16 (24.6)	29 (30.8)	
Connective tissue diseases	9 (13.89	21 (22.3)	
Fibromuscular dysplasia	13 (20.0)	17 (18.1)	
**Vascular lesion** (n, %)			
Present	656 60 (92.3)	86 (91.5)	0.54
Not present	5 (7.7)	8 (8.5)	
**Lesion type** (n, %)			
Dissection	26 (40.0)	34 (36.2)	0.07
Ectasia/aneurism	22 (33.8)	26 (27.6)	
Occlusion	10 (15.4)	10 (10.6)	
Stenosis	222 2 (3.1)	16 (17.0)	
No lesion	8 5 (7.7)	8 (8.5)	
**Vascular intervention** (n, %)			
Underwent intervention	161 16 (25.0)	34 (36.2)	0.16
No intervention	52 48 (75.0)	60 (63.8)	
**Hypertension** (n, %)			0.51
Yes	18 (27.7)	27 (28.7)	
No	47 (72.3)	67 (71.3)	00.8
**Total**	65 (40.9)	94 (59.1)	

Males were more likely to have a Z-score ≥ 2 compared to their female counterparts (33.8% vs 17%; *p* = 0.02). The two groups did not differ significantly in terms of age, hypertension status BMI, family history of rare vascular disease, genetic mutation**,** diagnosis, presence of arterial lesion and lesion type and vascular intervention.

Overall, the most frequently reported diagnosis was arterial dissection & aneurysm (33.9%), followed by arteritis & autoimmune disease (28.3%), connective tissue disease (18.9%), and FMD (18.9%). Dissection and aneurysm were the most frequent diagnosis among men (41.5%), whereas arteritis & autoimmune disease was the most frequent diagnosis among females (30.8%). Arterial lesions were detected in 146 patients (91.8%). Most frequently reported lesions were arterial dissections (37.7%), followed by arterial aneurisms (30.2%). A detailed description of lesion types across different diagnosis is reported in Additional file 1.

Vascular intervention was performed in 50 patients (31.6%). Females were more likely to undergo vascular intervention compared to males, although not significantly (females: 36.2 vs males: 25.0%). There was no difference in terms of intervention between the diagnosis groups.

Overall, considering cardiovascular risk factors, 45 patients (28.3%) were diagnosed with hypertension upon CTA. There was a difference in BMI across disease groups with arteritis & autoimmune diseases having the highest mean BMI (25.7) compared to other disease groups.

### Aortic tortuosity index

**Fig. 3:**
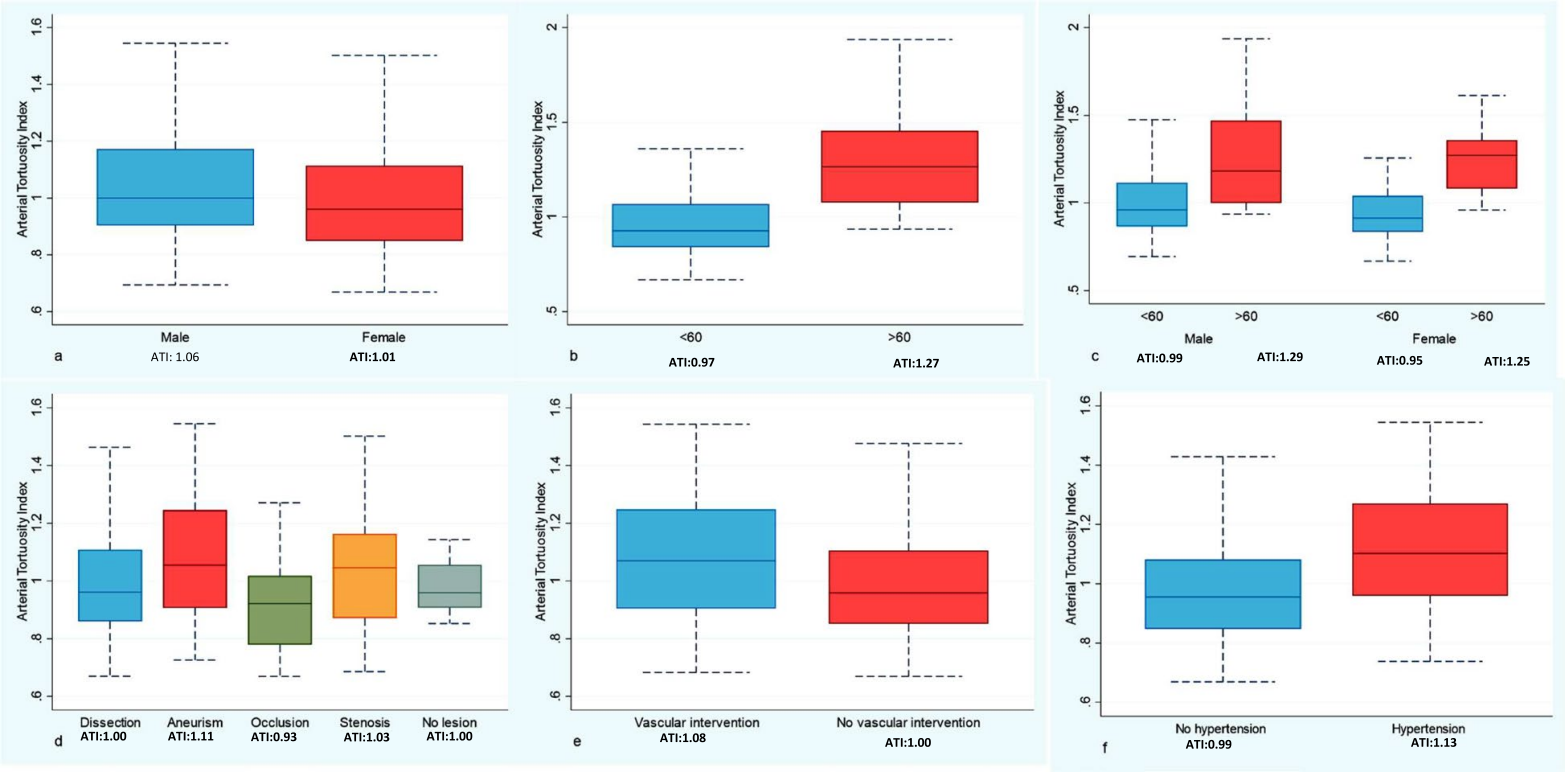
ATI values according to gender, age, hypertension status, lesion type and history of vascular intervention

**Fig. 4:**
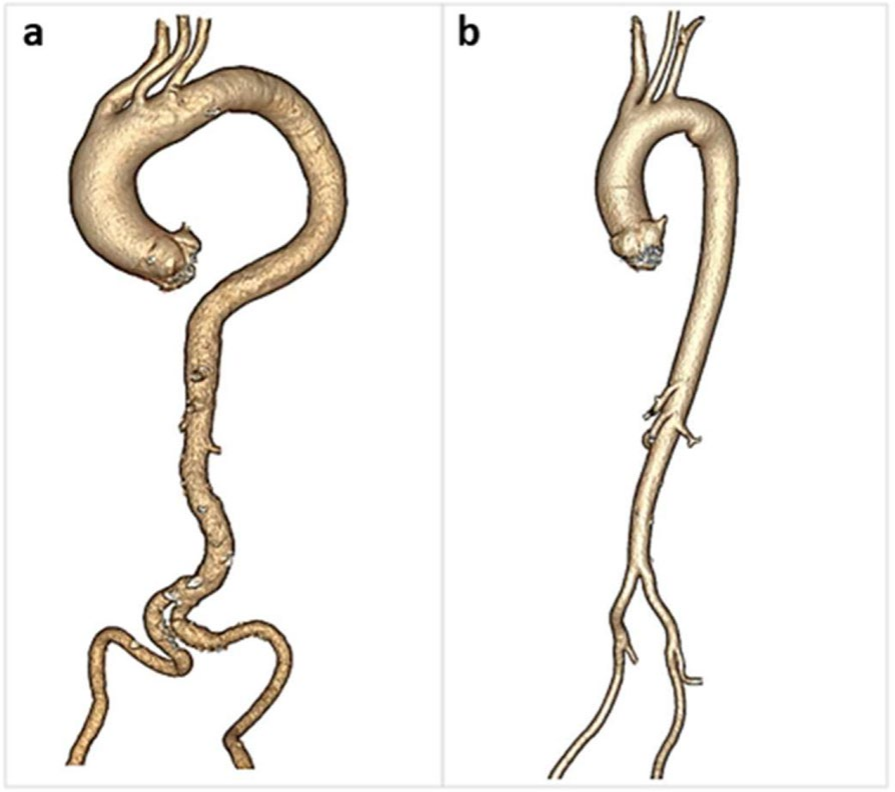
CTA scan of a 81-year-old male (**a**) diagnosed with arterial dissection & aneurysm with an ATI of 1.94 compared with a 55-year-old female (**b**) patient diagnosed with FMD with an ATI of 1.16

**Fig. 5:**
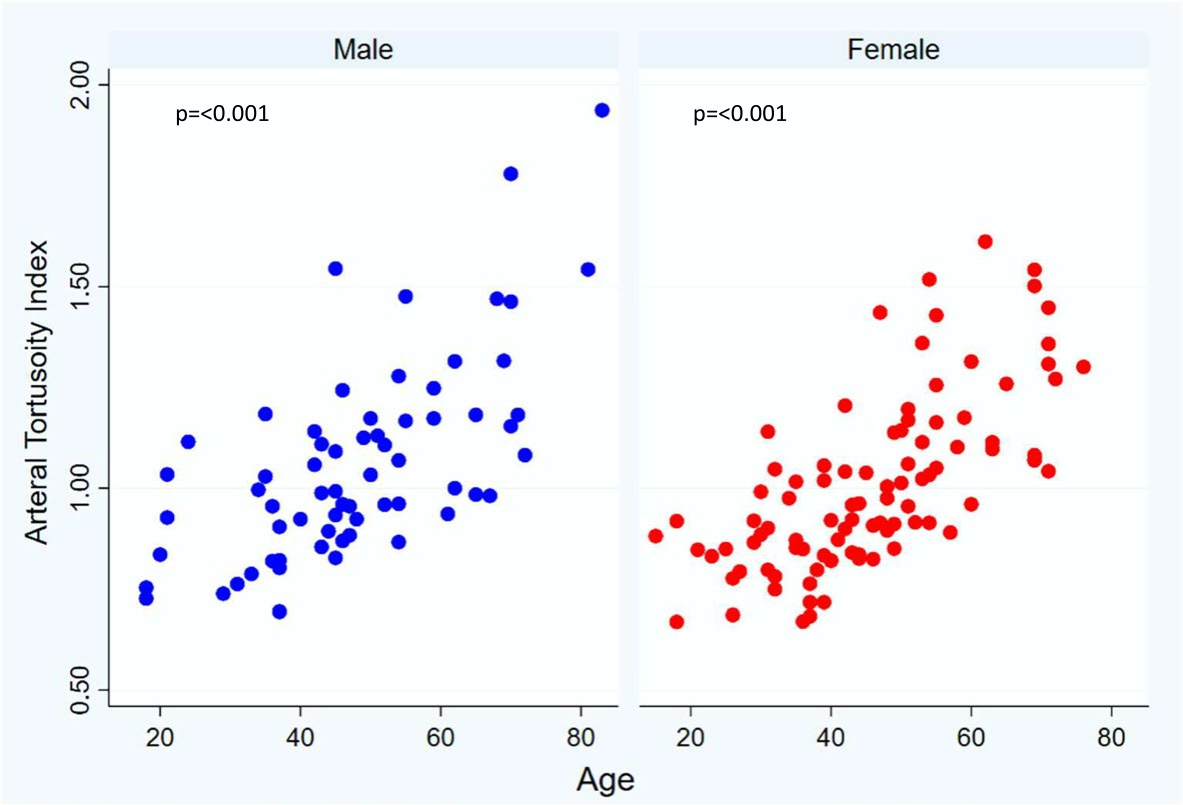
Arterial tortuosity index by age across males and female

Figure 3 shows ATI across gender, age, hypertension status, lesion type, and vascular intervention. Overall, average ATI was 1.03 (1.06 vs 1.00, males vs females, respectively) and increased with age (1.27 vs 0.97, ≥ 60 years old vs < 60 years old, respectively) in both males and females (Table 2, Figs. 4 and 5). Furthermore, mean ATI was higher in patients with arterial aneurysms as compared to other diseases groups (mean ATI:1.11) and in those diagnosed with hypertension (1.11 vs 0.99, hypertension vs no hypertension).

**Table 2:** Arterial Tortuosity Index across diagnosis split by gender and age

Mean ATI	All Mean: 1.03 (SD: 0.22, range 0.66–1.93	Gender		Age	
		**Male**	**Female**	** < 60**	** ≥ 60**
**Diagnosis**					
Arterial dissection & aneurysm	1.11	1.17	1.05	1.03	1.35
Arteritis & autoimmune disease	0.97	0.96	0.98	0.89	1.23
Connective tissue diseases	0.98	1.00	0.97	0.94	1.38
Fibromuscular dysplasia	1.02	1.01	1.03	1.00	1.09

The distribution of ATI in the study population is shown in Table 2.As for diagnosis, ATI was highest in patients with arterial dissection & aneurysm (mean: 1.11) and lowest in those with arteritis & autoimmune disease (mean: 0.97), with no significant differences between males and females. However, when only patients ≥ 60 years old were considered, those diagnosed with connective tissue diseases had the highest ATI (1.38), followed by patients with arterial dissection & aneurysm (mean:1.35) (Table 2).

### Predictors of aortic tortuosity

Multivariate analysis showed that ATI increased significantly with older age (*p* < 0.001). Patients diagnosed with aneurism lesions had significantly higher ATI (*p* < 0.001). ATI was also significantly higher among patients receiving anti-hypertensive medications compared to those receiving other medications (*p* < 0.03) (Table 3).

**Table 3:** Simple and multiple linear regression analyses showing factors associated with aortic tortuosity

Variables	Simple liner regression		Multiple linear regresion	
	**Exp (B)**	***p*-value**	**Exp (B)**	***p*-value**
Age	0.01	< 0.01	0.01	< 0.01
Sex	-0.05	0.13	-0.03	0.17
Body Mass Index	-0.01	0.07	-0.01	< 0.01
Ectasia/aneurism	0.10	0.02	0.12	< 0.01
**Hypertension**	**0.13**	** < 0.01**	**0.06**	** < 0.01**

## Discussion

In a heterogeneous cohort of patients referred to our center for suspected rare vascular disease, we found that aortic tortuosity increased with increasing age, whereas no difference was found between males and females. ATI was also significantly higher among subjects diagnosed with arterial dissection & aneurysm not associated with hereditary connective tissue disease, and particularly in those with aneurism lesions. However, when only patients over 60 years old were considered, those diagnosed with connective tissue diseases had the highest ATI values compared to other diseases. Mean ATI was higher among patients who were diagnosed with hypertension as compared to other patients. Our findings showed that increasing age, presence of aneurism lesions, and hypertension were independently associated with ATI. We observed that that lower BMI was associated with increased ATI.

Arterial tortuosity may be a marker of vascular fragility and a useful indicator of a series of vascular diseases, though the pathophysiological mechanisms underlying this phenomenon remain unclear. Several studies have focused so far on arterial elongation and tortuosity observed with aging, sex, and hypertension [3].

In fact, evidence on cervical arteries suggests that the tortuosity of vertebral arteries increases with age [27, 28], although the effect of aging on aortic tortuosity remains controversial. Previous research showed no correlation [29] or even an inverse correlation [30] between thoracic aorta tortuosity and aging. More recently, a study on 210 patients undergoing CTA showed that the length of the thoracic aorta significantly increased with increasing age in both males and females, whereas the tortuosity of the proximal descending aorta was only moderately associated with age [31]. In another study on patients without known vascular diseases, tortuosity of descending thoracic aorta was more pronounced in patients over 65 years old compared with younger ones [32]. In patients with genetically mediated aortic disease, the impact of age on arterial tortuosity is still unclear. Franken et al*.* calculated the ATI on magnetic resonance imaging in 211 patients with Marfan syndrome enrolled in the Dutch COMPARE trial and demonstrated a moderate but statistically significant correlation between ATI and age [20], which in line with our results seems to support the hypothesis that arterial tortuosity increases with age.

Several studies also suggest an association between arterial tortuosity and female sex. Martins et al*.* found that prevalence of internal carotid artery anomalies including kinking, coiling, and looping was higher in females than in males [33]. Similarly, two large studies on patients who underwent coronary angiography found a significant association between female sex and coronary artery tortuosity [34, 35]. Notwithstanding this, no difference was observed between the two sexes in terms of aortic tortuosity by others [32], as also highlighted by our study.

The association between arterial tortuosity and hypertension has been widely documented. Pancera et al*.* showed an association between arterial hypertension and kinking of the carotid artery in two cross-sectional studies [36, 37]. Li et al*.* found that hypertension was an independent predictor of coronary tortuosity in patients undergoing coronary angiography for chest pain [38]. Similarly, another study found that the hypertensive population has increased arterial tortuosity across multiple arteries [39]. Consistent with these findings, in our study, the presence of hypertension at the time of CTA was significantly and independently associated with aortic tortuosity. Interestingly, in the above study by Franken et al*.*, Losartan therapy had no effect on ATI, and use of Losartan was not associated with lower occurrence of dissection or the combined endpoint including prophylactic aortic surgery, aortic dissection, and death in patients with Marfan syndrome [20]. Further research will be needed to clarify the role of hypertension and anti-hypertensive medications in aortic tortuosity development in patients with and without rare vascular disease.

Our findings revealed a negative association between BMI and aortic tortuosity. In line with these, Moon et al*.,* reported negative linear correlation between aortic tortuosity and BMI [40]. A 2022 study showed a negative correlation between coronary artery tortuosity and BMI [41].

Arterial tortuosity is increased and indicates poorer prognosis in patients with heritable thoracic aortic disease [7]. Markedly increased tortuosity is prevalent in patients with Loeys-Dietz syndrome who develop early onset thoracic aortic dissections [19, 42]. In the above study by Franken et al*.*, the ATI was significantly higher in patients with Marfan syndrome than in healthy controls. The authors also demonstrated that higher ATI was not associated with faster aortic root growth over time but was strongly associated with occurrence of type B aortic dissection, as patients with an ATI > 1.95 had a more than 12-fold higher probability of meeting the combined endpoint and developing an aortic dissection at follow-up [20]. In a study on hypertensive adults without known genetically mediated disease, patients with aortic dissection were found to have longer aortas, greater aortic dimension, and volume, and greater arch tortuosity. However, when all these parameters were included in a multivariable model, arch tortuosity was only weakly predictive of dissection as compared to other anatomic factors [43]. In subjects with bicuspid aortic valve, increased aortic arch tortuosity was significantly associated with thoracic aortic disease [44]. Tortuosity of the aortic arch and descending thoracic aorta also predicted survival and aortic events in patients with type B dissection [43]. Lastly, Chen et al*.* found increased tortuosity of the ascending aorta in patients with acute type A aortic dissection than in healthy controls and demonstrated moderate correlation between ascending aortic dissection and tortuosity [45]. In our study, aortic tortuosity was significantly higher among patients with arterial dissection & aneurysm. Overall, these data highlight the potential prognostic role of aortic tortuosity that merits further investigation in larger prospective studies.

Along with stenosis, dissection, and aneurysm, arterial tortuosity is also part of the vascular spectrum of FMD. An association between tortuosity of the internal carotid artery and FMD was shown [46]. Furthermore, coronary angiography showed no significant coronary stenosis but marked coronary tortuosity in patients with resistant hypertension and chest pain [47]. However, the prevalence of aortic tortuosity may be lower in this population, as suggested by the relatively low values of ATI in patients with FMD in our study. Such aspect needs to be studied in larger cohorts of patients with this condition.

This study has several limitations worth mentioning. First, this is a small retrospective study with which limits the comparison of our results to the general population. Second, this was an observational study, therefore we cannot conclude that associations were causative [48]. Thirdly, our sample size was limited to 159 patients. Therefore, future studies with larger sample sizes are needed to confirm our findings.

Despite these limitations, our study shows some interesting aspects. In patients with a series of non-atherosclerotic vascular diseases, aortic tortuosity appears to increase with age and to be associated with the presence of arterial aneurysms and hypertension. The ATI may be a promising tool in diagnostic evaluation, cardiovascular risk stratification, medical or surgical management, and prognostic assessment of various vascular conditions [7, 18–21] including rare vascular diseases. However, incorporation of this arterial phenotype into clinical practice requires standardization in terms of definition, measurement, and normality criteria. Further studies with longitudinal design and larger cohorts are needed to validate the role of arterial tortuosity in the full spectrum of vascular disease.

## Supplementary Information

Supplementary Material 1.

## Data Availability

The data used and analyzed during the current study are not publicly available due to privacy and ethical restrictions. The data has been extracted from medical records owned by Lausanne University Hospital (CHUV), Switzerland at: https://www.chuv.ch/fr/rc/patients.
