# Correction: Tortuosity in non-atherosclerotic vascular diseases is associated with age, arterial aneurysms, and hypertension

**DOI:** 10.1186/s13023-025-03964-1

**Published:** 2025-09-01

**Authors:** Xhyljeta Luta, Fabio Zanchi, Marco Fresa, Enrica Porceddu, Sanjiv Keller, Judith Bouchardy, Sébastien Déglise, Salah Dine Qanadli, Matthias Kirsch, Grégoire Wuerzner, Andrea Superti-Furga, Giacomo Buso, Lucia Mazzolai

**Affiliations:** 1 Department of Angiology, University Hospital of Lausanne (CHUV), Lausanne, Switzerland; 2 Department of Radiology, University Hospital of Lausanne (CHUV), Lausanne, Switzerland; 3 Department of Cardiology, University Hospital of Lausanne (CHUV), Lausanne, Switzerland; 4 Department of Vascular Surgery, University Hospital of Lausanne (CHUV), Lausanne, Switzerland; 5 Department of Cardiac Surgery, University Hospital of Lausanne (CHUV), Lausanne, Switzerland; 6 Department of Nephrology and Hypertension, University Hospital of Lausanne (CHUV), Lausanne, Switzerland; 7 Department of Genetic Medicine, University Hospital of Lausanne (CHUV), Lausanne, Switzerland; 8 Riviera-Chablais Hospital, University of Lausanne, Lausanne, Switzerland

**Correction:** ***Orphanet J Rare Dis*** **19, 227 (2024)**

10.1186/s13023-024-03231-9

Following publication of the original article [1], the authors reported a typing error in the name of Enrica Porceddu. The corrected author’s name provided in this Correction is accurate.

The original article [1] has been updated.

## References

[1] Luta X, Zanchi F, Fresa M, et al. Tortuosity in non-atherosclerotic vascular diseases is associated with age, arterial aneurysms, and hypertension. Orphanet J Rare Dis. 2024;19:227. 10.1186/s13023-024-03231-9. 38849913 10.1186/s13023-024-03231-9 PMC11157772

