# Supplementary material for: Tortuosity in non-atherosclerotic vascular diseases is associated with age, arterial aneurysms, and hypertension
Source: Orphanet J Rare Dis. 2024 Jun 7;19:227. doi: 10.1186/s13023-024-03231-9 (PMC11157772; doi:10.1186/s13023-024-03231-9)
Supplement: Supplementary file 1 — Supplementary Material 1. [file 13023_2024_3231_MOESM1_ESM.docx]

**Supplementary file 1:** Lesion types across diagnosis

| **Clinical characteristics** | **Lesion type** | | | | | |
| --- | --- | --- | --- | --- | --- | --- |
| **Diagnosis** | **Dissection** | **Aneurism** | **Occlusion** | **Stenosis** | **No lesion** | **Total** |
| Arterial dissection & aneurysm | 31 (50.0) | 20 (40.8) | 1 (5.0) | 1 (5.6) | 1 (5.8) | 54 (32.5) |
| Arteritis & autoimmune disease | 0 (0.0) | 7 (14.3) | 18 (90.0) | 12 (66.7) | 8 (47.1) | 45 (27.1) |
| Connective tissue diseases | 11 (17.7) | 19 (38.8) | 0 (0.0) | 0 (0.0) | 7 (41.2) | 37 (22.3) |
| Fibromuscular dysplasia | 20 (32.3) | 3 (6.1) | 1 (5.0) | 5 (27.8) | 1 (5.8) | 30 (18.1) |
| Total | 62 (100) | 49 (100) | 20 (100) | 18 (100) | 17 (100) | 166 (100) |
